# External quality assessment (EQA) program for the immunohistochemical detection of ER, PR and Ki-67 in breast cancer: results of an interlaboratory reproducibility ring study in China

**DOI:** 10.1186/s12885-019-6210-3

**Published:** 2019-10-22

**Authors:** Tianjie Pu, Ruohong Shui, Jie Shi, Zhiyong Liang, Wentao Yang, Hong Bu, Qin Li, Zhang Zhang, China Anticancer Association Professional Committee of Tumour Pathology, Deyu Guo, Bo Huang, Fangping Xu, Yun Ma, Jiping Qi, Qiurong Ruan, Yang Weng, Danhua Shen, Xiaomei Li, Yunte Deng, Julun Yang, Lixia Wang, Xianghong Yang, Rong Yang, Yueping Liu, Lingfei Kong, Peng Gao, Fang Mei, Xiu Nie, Min Yao, Wei Qu, Chuansheng Huang, Mei Liu, Mumin Shao, Zhihong Zhang, Jiehua He, Huaisheng Lv, Huixiang Li, Xianglei He, Shuangping Guo, Weicheng Xue, Linying Chen, Jingping Yuan, Yonghong Shi, Qing Sun, Weiqiang Zheng, Wenyong Sun, Fan Zhang, Yunjie Zeng, Wei Zhang, Chenggang Yang

**Affiliations:** 1 Department of Pathology, West China Hospital, Sichuan University, Guo Xue Xiang 37#, Chengdu, 610041 Sichuan China; 2 Laboratory of Pathology, West China Hospital, Sichuan University, Chengdu, Sichuan China; 3 Department of Pathology, Shanghai Cancer Center, Fudan University, Shanghai, China; 4 Department of Pathology, Peking Union Medical College Hospital, China Academy of Medical Science and Peking Union Medical College, Beijing, China; 5 Department of Hospital Infection Control, Women’s and Children’s Hospital of Sichuan Province, Chengdu, China

**Keywords:** Breast neoplasm, Immunohistochemistry, Quality control, Estrogen receptors, Progesterone receptors, Ki-67 antigen

## Abstract

**Background:**

An External Quality Assessment (EQA) program was developed to investigate the status of estrogen receptor (ER), progesterone receptor (PR), and Ki-67 immunohistochemical (IHC) detection in breast cancer and to evaluate the reproducibility of staining and interpretation in 44 pathology laboratories in China.

**Methods:**

This program was implemented through three specific steps. In study I, three revising centres defined the reference value for 11 sections. In study II, 41 participating centres (PC) stained and interpreted 11 sections by their own daily practice IHC protocols. In study III, all cases received second interpretation opinions.

**Results:**

The stained slides of 44 laboratories were up to the interpretation standard. The overall interpretation concordance rate of this study was over 90%. A perfect agreement was reached among the PCs for the cases with ER+ and PR+ > 50% and Ki-67 > 30%, whereas a moderate agreement was observed for intermediate categories. After second interpretations, the misclassification rates for ER were reduced by 12.20%, for PR were reduced by 17.07%, and for Ki-67 were reduced by 4.88%. Up to 31 PCs observed a benefit from the second opinion strategy.

**Conclusions:**

This project is the first EQA study performed on a national scale for assessment of ER, PR and Ki-67 status by IHC in China. In the whole IHC evaluation process, the intermediate categories were less reproducible than those with high expression rates. Second opinions can significantly improve the diagnostic agreement of pathologists’ interpretations.

## Background

Breast cancer (BC) survival has improved by approximately 25% over the past two decades [1]. This improvement is due, in part, to advances in the understanding of breast cancer pathogenesis and targeted therapies. There is an almost worldwide acceptance that the measurement of estrogen receptor (ER), progesterone receptor (PR), human epidermal growth factor receptor 2 (HER-2) and Ki-67 status provides valuable information to aid in the selection of patients who would benefit from endocrine treatment, targeted agents and chemotherapy. Therefore, it is the pathologist’s responsibility to assure accurate and reliable assessment of expression of breast cancer biomarkers [2, 3]. Among all the different methods used in routine clinical practice, immunohistochemistry (IHC) is the most commonly used, with extensive validation by international guidelines [4].

However, IHC tests, including ER, PR, HER2 and Ki-67 tests, have historically suffered from poor reproducibility [5–7]. This is well illustrated by the studies of Rhodes et al. [8], McCullough et al. [9] and Niikura et al. [10], who showed that the main problems in detection of biomarkers are technically suboptimal protocols and the assessment of results.

External quality assessment (EQA)-a system that retrospectively and objectively compares staining results from many laboratories by means of an external agency, allows the identification of insufficient stains and inappropriate protocols, as well as the identification of possible interpretation problems [11, 12]. An EQA could serve as an early warning system for potential problems and as an indicator of where to direct improvement efforts and identify training needs. Therefore, an EQA should be implemented in clinical immunohistochemistry laboratories.

In the past 5 years, EQA of HER2-IHC in breast cancers in China has been performed by the Pathology Quality Control Centre (PQCC) of the National Health and Family Planning Commission with the aim of assessing consistency and accuracy regarding HER2-IHC in different pathology departments. However, the data regarding IHC for ER, PR and Ki-67 were sparse. In this context, we performed a three-step EQA study for assessment of ER, PR and Ki-67 protocols in order to evaluate their accuracy related to both the staining and interpretation of IHC assays. This paper reports the results of this EQA program to demonstrate the current status of breast cancer-associated IHC detection in China.

## Methods

This study was approved by China Anticancer Association Professional Committee of Tumour Pathology.

### Study design

**Fig. 1:**
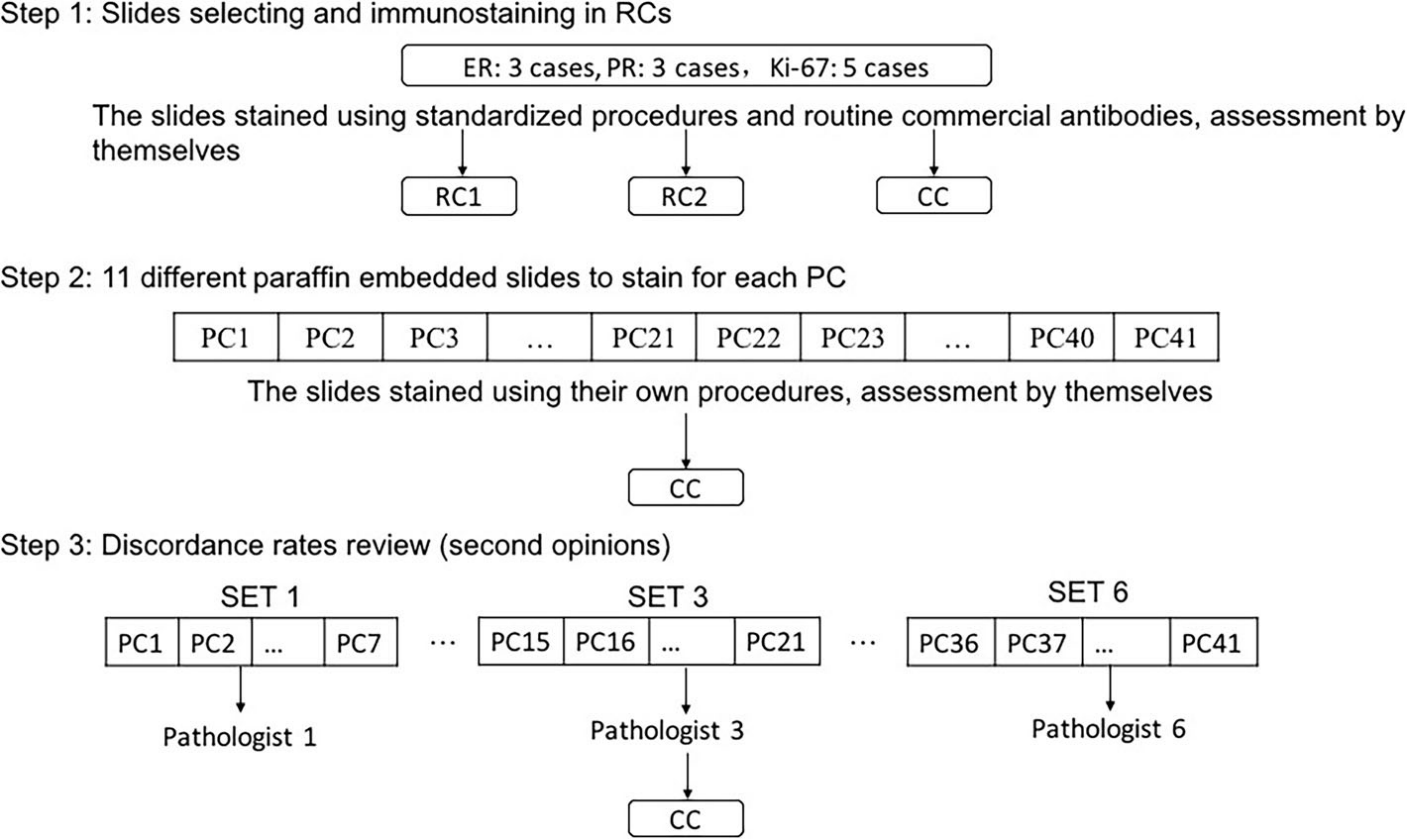
Workflow of the EQA program

This EQA program was implemented via 3 specific studies (Fig. 1). Study I and II were designed to examine interinstitutional consistency. Study III was designed to examine interobserver consistency. The management activities of this program were assigned to different working units: the coordinating centre (CC), the revising centres (RCs) and the participating centres (PCs).

For study I, the RCs stained the slides by standardized procedures using three kinds of antibodies, and more details showed in Additional file 1: Table S1. Tests for ER utilized the monoclonal antibodies EP1 (Dako, Glostrup, Denmark), SP1 (Ventana, Tucson, Arizona, USA) and 6F11 (Leica, Bannockburn, IL). Tests for PR utilized the monoclonal antibodies; PgR636 (Dako, Glostrup, Denmark), 1E2 (Ventana, Tucson, Arizona, USA) and 16 (Leica, Bannockburn, IL). In tests for Ki-67, the following monoclonal antibodies were utilized: 30–9 (Ventana, Tucson, Arizona, USA), K2 (Leica, Bannockburn, IL) and MIB-1 (Maxim, China). Three sets of 11 BC sections were sent to each RC for testing and optimization of the different antibodies until all RCs obtained the same IHC results.

For study II, each PC received a set of 11 BC sections. All PCs filled out a questionnaire before the start of the study in order to gather information regarding their routine methods in determining the status of ER, PR and Ki-67. Each PC stained these slides by adopting their own procedures and then sent the 11 slides and their interpretation back to the CC. To test the accuracy of the PCs immunohistochemical techniques, the 11 staining slides were sent back to the CC, the pathologist of CC tested whether the control tissues were stained correctly, and then he/she reviewed 11 sections from each PC. We also pay attention to whether there was a significant difference of the percentage of staining among PCs and the agreement between the results and reference values.

To answer the question of the accuracy of the PCs interpretation, we setup study III. We randomly assigned all slides from 41 PCs to 6 testing sets and delivered them to 12 experienced pathologists (that is, committee members of the PQCC and RCs) in a blinded manner. As a secondary analysis, the agreement rates between assessments of the same case by PC and second opinions represented the level of interpretation of the PC. The results of this study were analysed by an independent coordinator, who had no relationship with or role at any of the reference centres, after completion of all testing rounds.

### Participants

We recruited 44 pathology laboratories all around China in this EQA program according to the following criteria: 1) over 150 detected cases/yr of IHC- positive breast cancer, 2) participation in PQCC testing training, and 3) possession and implementation of internal standard operating procedures (SOPs).

The CC (Department of Pathology, West China Hospital, Sichuan University, China) is the PQCC of West China. The CC that coordinated the logistical and practical aspects of the EQA collected a series of ER-, PR-, and Ki-67-positive and ER-, PR-, and Ki-67-negative BC cases from its own tissue sample archive. Two RCs, the Department of Pathology of Peking Union Medical College Hospital and the Shanghai Cancer Centre of Fudan University, PQCC of North and East China, together with the CC, contributed to selecting the BC slides to be included in the EQA and to defining the reference value.

### Sample selection and distribution

**Fig. 2:**
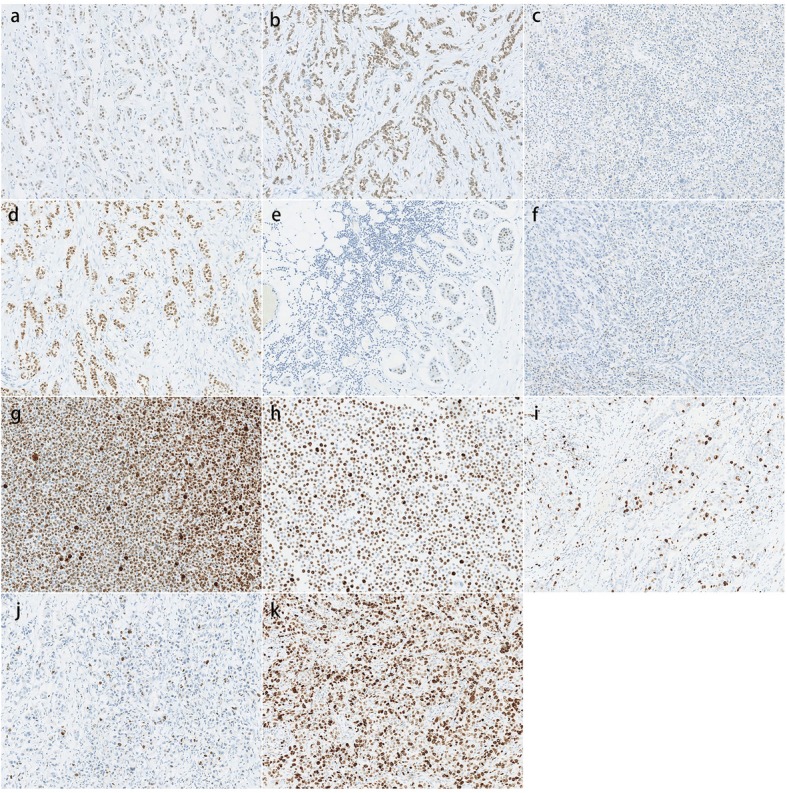
Optimal staining for ER, PR and Ki-67 that was deemed to be the reference value. **a** ER-1: 11–50% positive staining (× 100). **b** ER-2: > 50% positive staining (× 100). **c** ER-3: negative staining (× 100). **d** PR-1: > 50% positive staining (× 100). **e** PR-2: 1–10% positive staining (× 100). **f** PR-3: negative staining. **g-h** KI-1and KI-2: > 30% positive staining in breast cell lines (MCF-7 and MDA-MB-231, × 100). **i-k** Ki-67 staining in breast carcinoma tissue. **i** KI-3: 10–30% positive staining (× 100). **j** KI-4: < 10% positive staining (× 100). **k** KI-4: > 30% positive staining (× 100). ER: estrogen receptor, PR: progesterone receptor

This study used “in house” sections, all derived from the CC, to exclude variable factors in sample procedures (e.g., fixation of tumour samples, absorbance, and tissue embedding) [13]. All of the specimens had been fixed with formalin (12 h) and embedded in paraffin blocks. To simulate the routine assessment in clinical laboratories, we used whole blocks from surgical pathology specimens, possibly providing more areas of heterogeneity, instead of tissue microarrays, which are useful for analysing large numbers of samples [14, 15]. In total, 11 specimens (3 for ER, 3 for PR and 5 for Ki-67) of invasive breast cancer had been previously tested for ER, PR and Ki-67 status by immunohistochemistry, and these specimens were requested to represent a range of immunohistochemical expression levels (Fig. 2). Each block provided 46 consecutive sections. The CC performed staining on the first and last sections to ensure that positively stained cells were present for analysis on each slide [16].

The sections from 11 specimens containing normal breast tissue that were used as internal controls to determine whether the IHC staining was working.

### Assessment of slides

The proportion of positively labelled to unlabelled tumour nuclei was counted, disregarding the intensity of the reaction [17, 18]. Immunohistochemical specimens for ER and PR evaluation procedure were selected according to the ASCO-CAP guidelines [4]. Scoring was done on a point scale. Immunohistochemistry specimens for ER and PR were scored by the proportion of positive staining tumor nuclei, as 0, < 1, 1–10%, 11–50%, > 50%.

For Ki-67 staining, the whole slide was scanned under low-power microscopy first. At least three high-power (40x objective) fields were selected in hot spots [19], which were defined as areas in which Ki-67 staining was the densest among the fields. Then, the pathologists counted 1000 cells, with 500 cells as the absolute minimum [20, 21], and the positivity rate was calculated and classified into four groups: 0, < 10, 10–30%, > 30%.

Appropriate control specimens were also tested.

### Statistics

The performance of each PC was evaluated by comparing their own interpretation of the slides with the reference values, and the agreement rate and intraclass correlation coefficient (ICC) were calculated with a 95% confidence interval (CI). Higher ICC usually indicates better consistency. There is no universally accepted standard criteria for the ICC; based on the similarity to the kappa coefficient, 0.00–0.20 was interpreted as “slight correlation”; 0.21–0.40, as “fair correlation”; 0.41–0.60, as “moderate correlation”; 0.61–0.80, as “substantial correlation”; and > 0.80, as “almost perfect correlation” [20, 22]. The agreement rate between the initial pathologist’s diagnosis and the second pathologist’s diagnosis was estimated.

Statistical analyses were performed with SPSS (Version 22.0; SPSS Inc., Chicago, USA).

## Results

### Study I

All the RCs stained the slides by standardized protocols using three commercial validation antibodies. As all RCs obtained the same results, the proportions of tumour nuclei positive for ER-1, ER-2 and ER-3 were 11–50%, > 50 and 0%, respectively. For the PR tests, the reference values were > 50% for PR-1, 1–10% for PR-2 and 0% for PR-3. For the Ki-67 tests, the reference values were > 30% for KI-1, KI-2 and KI-5; 10–30% for KI-3; and < 10% for KI-4 (Additional file 1: Figure S1).

### Study II

The results of the questionnaire are reported in Table 1. The frequency distribution of the responses indicated methodological heterogeneity among the 41 laboratories. All the PCs used the DAB chromogen in their protocols. Only 7 PCs used a manual immunostaining protocol. The monoclonal antibody SP1 (Ventana, Tucson, Arizona, USA) was the most commonly used reagent for the ER test; 1E2 (Ventana, Tucson, Arizona, USA), for the PR test; and MIB-1(Maxim, China), for the Ki-67 test. The majority of PCs used a heat retrieval step in an automated immunostainer.

**Table 1:** Questionnaire results from the 41 participant centres

	ER, N (%)		PR, N (%)		Ki-67, N (%)
Immunostaining procedure					
Automated	34 (82.9)		34 (82.9)		34 (82.9)
Manual	7 (17.1)		7 (17.1)		7 (17.1)
Type of antibody					
SP1(Ventana)	26 (63.4)	1E2(Ventana)	19 (46.3)	30–9(Ventana)	12 (29.3)
EP1(Dako)	10 (24.4)	EP2(BIO-SB)	9 (22.0)	MIB-1(Maxim)	14 (34.1)
6F11(Leica)	2 (4.9)	PgR36(Dako)	4 (9.7)	UMAB107(Origene)	7 (17.1)
others	3 (7.3)	others	9 (22.0)	others	8 (19.5)
Antigen retrieval					
Automated	33 (80.5)		33 (80.5)		33 (80.5)
Pressure cookers	8 (19.5)		8 (19.5)		8 (19.5)
Chromogen					
DAB	41 (100)		41 (100)		41 (100)
Evaluation					
Pathologist	41 (100)		41 (100)		41 (100)
Artificial intelligence	0		0		0

**Fig. 3:**
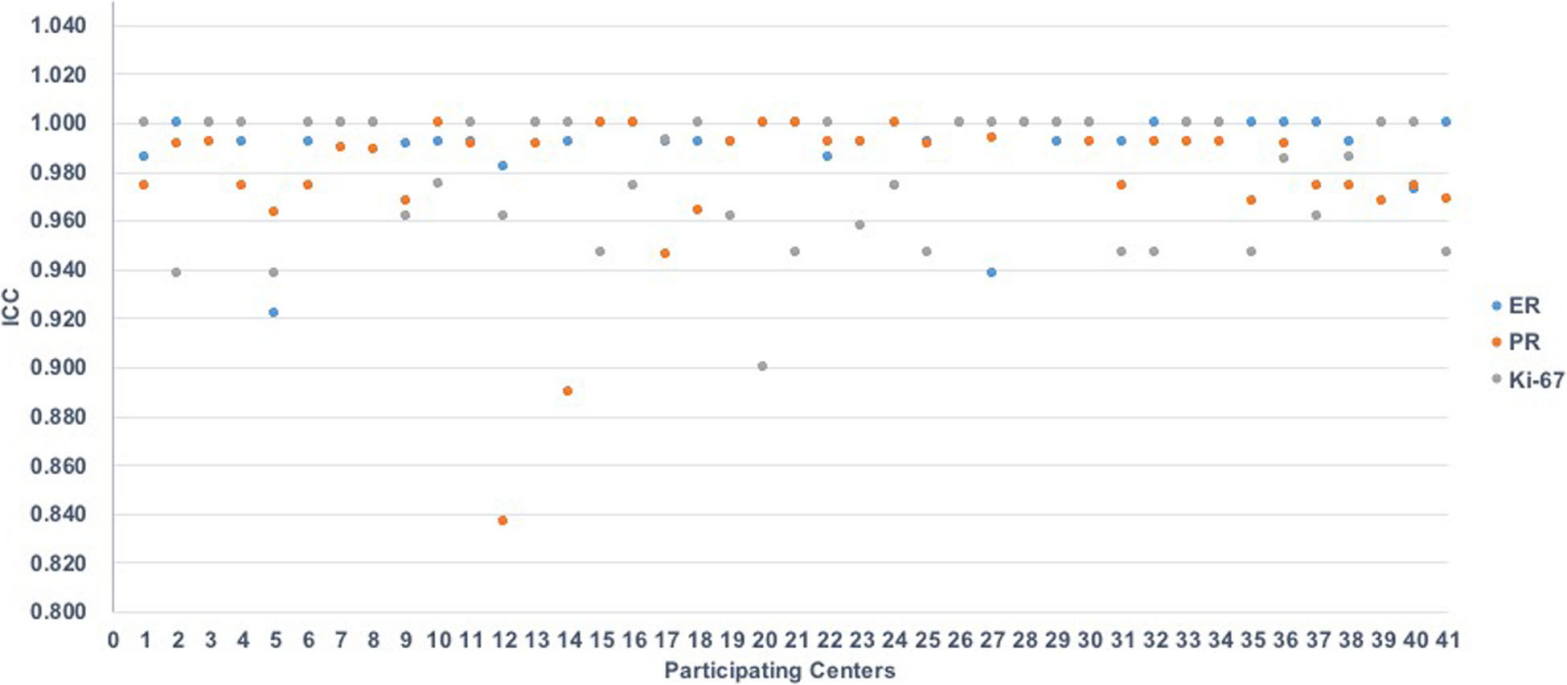
Summarization of intraclass correlation coefficient (ICC) values for ER, PR, and Ki-67. ER: estrogen receptor, PR: progesterone receptor

The performance of each PC was evaluated by comparing their own interpretation of the stained slides with the reference values using the intraclass correlation coefficient (ICC) (Fig. 3). A better correlation was demonstrated for ER than for Ki-67 (ICC: 0.987 and 95% CI: 0.964–0.998 for Ki-67; ICC: 0.998 and 95% CI: 0.994–1 for ER). The ICC of PR demonstrated a correlation (ICC: 0.997; 95% CI: 0.99–1), between ER and Ki-67.

**Fig. 4:**
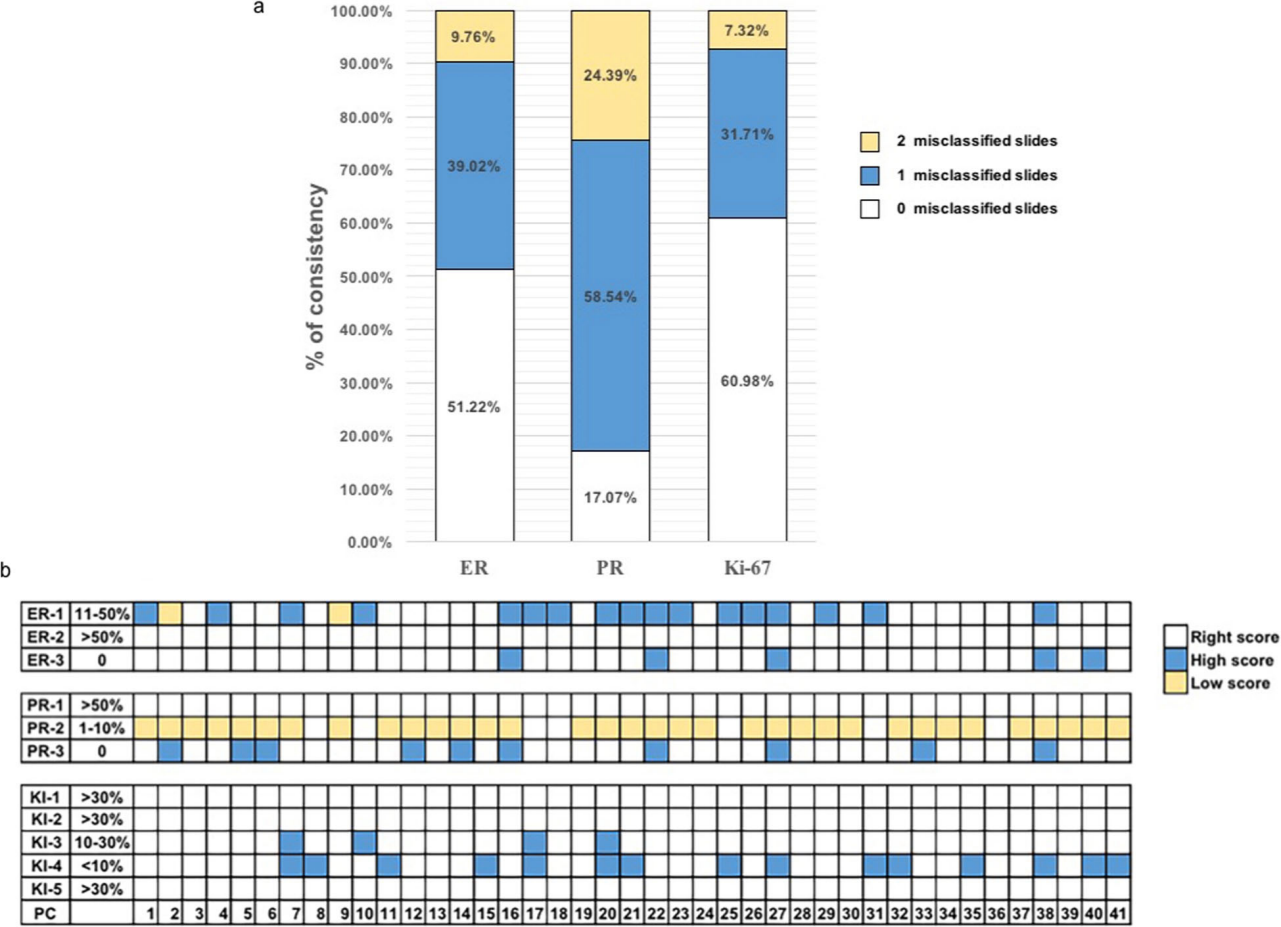
Interpretation of ER, PR, and Ki-67 immunostaining results in 41 PCs. **a** The misclassification rate compared to the reference values (Total N° of misclassified slides). **b** The misclassifications cases compared to the reference values. PCs: participant centres

In regard to ER immunostaining, all the slides were correctly immunostained in 21 PCs (21/41, 51.22%). In total, 16 PCs (16/41, 39.02%) provided 2 out of 3 slides in accordance with the reference value. For the remaining 4 PCs (4/41, 9.76%), the correspondence between their results and reference value was found for 1 out of 3 slides (Fig. 4a). All of the PCs gave a correct immunostaining result for ER-2 (> 50%). Nineteen immunostained slides did not correspond to ER-1 (11–50%); among these, 17/19 slides were > 50%, and 2/19 were identified as 1–10%. Concerning ER-3 (0%), 3/5 of them were given < 1%, and 2/5 of them were considered 1–10% (Fig. 4b).

The observed agreement for PR staining was lower (7/41, 17.07%). Twenty-four PCs (24/41, 58.54%) provided 1 discordant value out of 3, and 10 PCs (10/41, 24.39%) provided only 1 out of 3 slides in accordance with the reference value (Fig. 4a). It is worth noting that no PR-1 (> 50%) was misclassified. Conversely, we observed 34 and 10 misclassifications in PR-2 (1–10%) and PR-3 (0%), respectively. For PR-2 (1–10%), 18/34 slides were misclassified as <1%, and 16/34 slides were interpreted as 0%. Concerning PR-3 (0%), 7/10 slides were interpreted as <1%, and 3/10 slides were over interpreted (1–10%) (Fig. 4b).

The observed agreement for Ki-67 staining was good (25/41, 60.98%). Thirteen PCs (13/41, 31.70%) provided 4 out of 5 slides in accordance with the reference value. For the other 3 PCs (3/41, 7.32%), the correspondence between their interpretation result and the reference value was found to be 3 out of 5 slides (Fig. 4a). High expression of Ki-67 yielded the highest interlaboratory concordance. Finally, concerning KI-3 (10–30%), 4 slides were not immunostained properly, and all of them were interpreted as > 30%. We observed that 15 slides of KI-4 (< 10%) were misclassified as 10–30%. For KI-1, KI-2 and KI-5 (> 30%), there were no misclassified slides (Fig. 4b).

### Study III

As a secondary analysis, we evaluated the agreement rates between assessments of the same case by single readers and second opinions. The average of the agreement between single interpretations and reference scores was 80.93%, whereas the corresponding agreement rate for interpretations that included second opinions was 90.91%.

The highest misclassification rate within diagnostic categories after single interpretation was for cases of PR (35.77%), followed by ER (19.51%) and Ki-67 (8.78%). After second interpretations, the misclassification rates for ER were reduced by 12.20%, for PR were reduced by 17.07%, and for Ki-67 were reduced by 4.88%. In particular, for PR-2, the misclassification rate was 82.93% for the single opinion, but it was reduced to 46.34% after the second opinion.

**Fig. 5:**
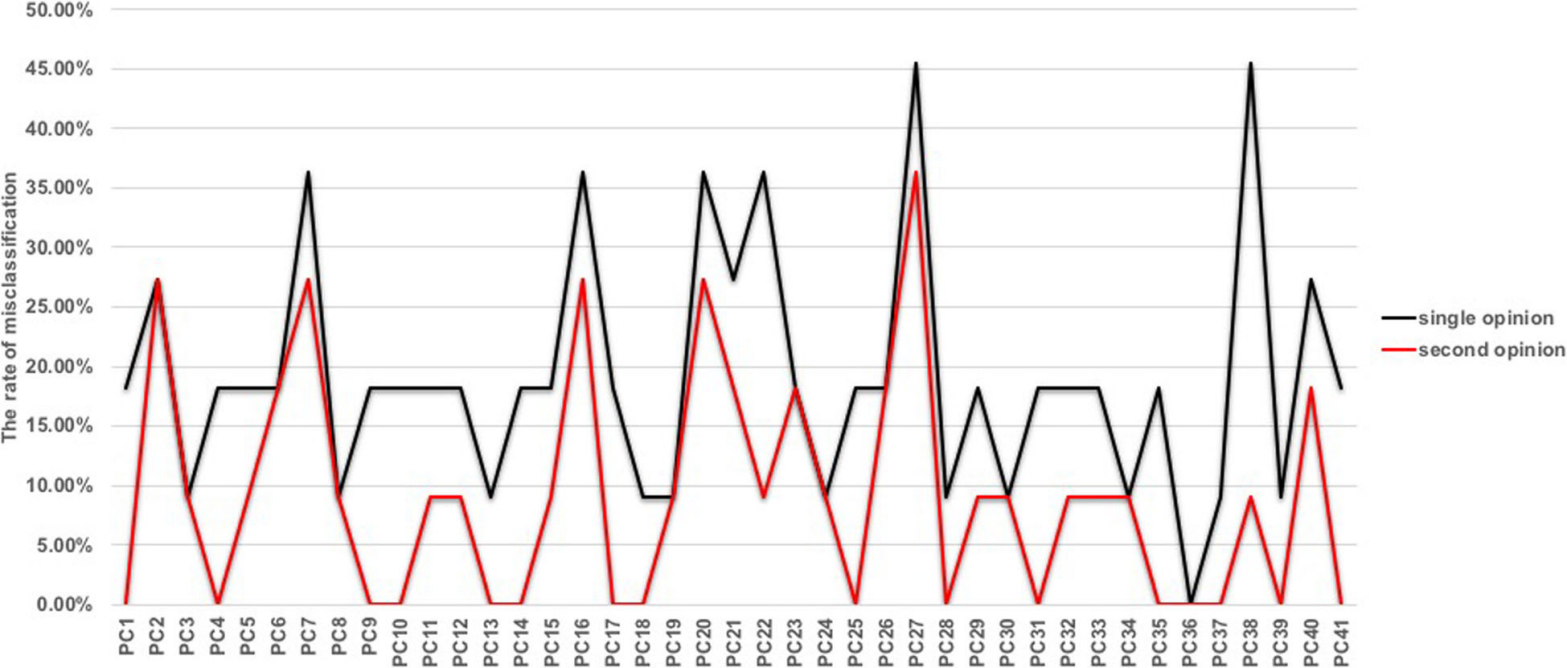
The rate of interpretation misclassification for the 41 PCs for single opinions (black line) and for second opinions (red line). PCs: participant centres

Up to 31 PCs benefited from the second opinion strategy. In particular, the misclassification rates of PC38 were reduced by 36.36% after the second interpretation (Fig. 5).

## Discussion

Since the EQA of HER2-IHC in breast cancers was a major project of the PQCC lasting for about 5 years, the detection and quality control of other biomarkers are also a work in progress. Here, we report on the largest study to date evaluating interlaboratory and interobserver agreement on semiquantitative IHC assessment of ER, PR and Ki-67 by ordinary clinical practice in China. The results are based on the evaluation of 11 slides stained by 44 participating laboratories across the country. Our three-step EQA study had a high concordance rate (> 90%) of IHC assessment for these biomarkers.

Semiquantitative IHC assessment of ER and PR was used as one of the main criteria to predict the likelihood of response to endocrine treatment in breast carcinoma. The ASCO/CAP guidelines recommend a specimen to be considered positive if 1% of the invasive tumour cells are positively stained [4]. In regard to the EQA, 2/41 and 3/41 PCs misclassified 0% as 1–10%, which would be classified as positive for ER-3 and PR-3, respectively. For these slides, there was weak cytoplasmic staining in the tumour cells. Pathologists who had less clinical experience interpreted the results as positive. This would lead the patient to receive ineffective endocrine therapy. Regarding PR-2, 34 PCs misclassified 1–10% as 0%. Small populations of positive cells were ignored during interpretation. This would exclude potentially eligible patients from the correct therapy regimen.

The observed agreement across PCs showed a good level of standardization of IHC procedures between each laboratory for ER-2 and PR-1 (> 50%), both for the immunostaining and for the interpretation. Discordant results mostly occurred in the ER-1(11–50%) and PR-2 (1–10%), emphasizing the level of subjectivity in evaluation of reproducibility of the intermediate scoring categories. The second opinion strategy [23] and computerized digital image analysis could be particularly useful to bring objective and accurate biomarker quantification for these difficult cases.

Currently, there are no standard methods to assess Ki-67 expression in breast cancer. Biological heterogeneity of Ki-67 staining can occur across breast cancer specimens. Differences in cell numbers which are counted and the selection of different tumor areas that should be scored are controversial and have been important reasons for the low interobserver reproducibility [24]. Hida’s study showed that “grey zone” categories (10–20%) are generally less reproducible than low- and high-value categories [25]. In our study, we classified IHC results of Ki67 into four groups: 0%, ≤10, 11–30%, and > 30% [26, 27] to avoid the “grey zone”. Therefore, the agreement between Ki-67 staining was good (25/41, 60.98%). We observed 4 slides upper-classified in relation to the KI-3 reference of 10–30%, and 15 slides upper-classified observed in KI-4 (< 10%), which may erroneously identify a potentially eligible patient for therapy, as the Ki-67 index of 20–30% is the boundary value for making clinical decisions. Further study should focus on IHC results of Ki67 including “grey zone” categories.

In our study, the second opinion strategy showed statistically significant improvements in accuracy. We had pathologists with high clinical volumes provide second opinions. The rates for overall misclassification decreased up to 36.36% when second opinions were obtained (PC38). Misclassification rates for single readings were higher for cases that were classified as borderline or difficult; however, these rates were also reduced when a second opinion was obtained (PR-2: 82.93% for the single opinion, 46.34% for the second opinion). In actual clinical practice, obtaining second opinions in such diagnostically complex areas might promote, over time, consensus within practices by highlighting diagnostic areas requiring education or expert consultation. If the second opinions came from less experienced pathologists, then the results might look very different and lead to misclassification. Therefore, this is a potential strategy to address the computerized digital image analysis. Yet, despite evidence that image analysis improved IHC biomarker scoring accuracy and reproducibility in tumors [28, 29], the adoption of computer-aided diagnosis by pathologists had remained limited in daily practice in China, especially based on heavy workload and low price of surgical specimens. This can be explained by the surplus of time required to correctly identified of tissue compartments relevant for assessment, correct morphology (normal vs in situ vs invasive) and stromal stain vs tumor stain, and by the difficult identified nuclei or membranes [30, 31].

At the end of this EQA, we provided the results to the participating units and found that a large number of laboratories would probably benefit greatly from participation in such programs. A variety of antibodies were used in different PCs in this study, which may be one of the reasons why the interpretations were not consistent. Therefore, future work should focus on promoting the use of a standard operating system (antibody type, staining process and interpretation standard), introducing educational programs, increasing the number of cases analysed and continuing enrolment of laboratories to increase the feasibility of implementing an EQA and making the process of IHC more standardized and accurate.

## Conclusions

We assessed the quality and consistency of ER, PR and Ki-67 testing by comparing interinstitutional and interobserver results on a national scale. The overall concordance rate of this study was over 90%. The results of this study suggest that the detection of biomarkers by IHC can be used for clinical treatment decisions. We strongly believe that EQA programs have the potential to improve our diagnostic precision and patients’ care. Participating in these programs is essential for achieving and maintaining the highest standard of care for breast cancer patients.

## Supplementary information

**Additional file 1: Table S1**. Standardized IHC staining procedures of RCs. **Figure S1**. Observed agreement between 3 RCs and the reference value. Three RCs, the Department of Pathology, West China Hospital, Sichuan University, the Department of Pathology of Peking Union Medical College Hospital and the Shanghai Cancer Center of Fudan University, stained the slides by standardized procedures using three kinds of antibodies. As all RCs obtained the same results, the proportions of tumour nuclei positive for ER-1, ER-2 and ER-3 were 11–50%, > 50 and 0%, respectively. For the PR tests, the reference values were > 50% for PR-1, 1–10% for PR-2 and 0% for PR-3. For the Ki-67 tests, the reference values were > 30% for KI-1, KI-2 and KI-5; 10–30% for KI-3; and < 10% for KI-4. RCs: revising centres

## Data Availability

All data generated or analyzed during this study are included in this published article or are available from the corresponding author on reasonable request.

## Abbreviations

BC: Breast cancer
CCs: Coordinating centre
CI: Confidence interval
EQA: External Quality Assessment
ER: Estrogen receptor;
HER-2: Epidermal growth factor receptor 2
ICC: Intraclass correlation coefficient
IHC: Immunohistochemistry
PCs: Participating centres
PQCC: Pathology Quality Control Centre
PR: Progesterone receptor
RCs: Revising centres
