# Supplementary material for: External quality assessment (EQA) program for the immunohistochemical detection of ER, PR and Ki-67 in breast cancer: results of an interlaboratory reproducibility ring study in China
Source: BMC Cancer. 2019 Oct 22;19:978. doi: 10.1186/s12885-019-6210-3 (PMC6805628; doi:10.1186/s12885-019-6210-3)

**Supplementary Table 1** Standardized IHC staining procedures of RCs

|  | Monoclonal Primary Antibody (Clone) | | | Incubation | Immunostainer | Antigen Retrieval | 2nd antibody | Incubation | Solution of chromoge | Incubation |
| --- | --- | --- | --- | --- | --- | --- | --- | --- | --- | --- |
| ER | Ventana | SP1 | RTU | 37℃ x 24min | BenchMark Ultra | CC1（pH8.2） x 36min | Ventana Multimer | 37℃ x 8min | DAB | 37℃ x 8min |
|  | DAKO | EP1 | RTU | 37℃ x 36min | DAKO Omnis | Target Retrieval Solution (pH6.1) x 64min | DAKO Envision+ Dual link system-HRP | 37℃ x 20min | DAB | 37℃ x 10min |
|  | Leica | 6F11 | RTU | 37℃ x 15min | Leica Bond III | ER2 x 25min | Leica Polymer | 37℃ x 8min | DAB | 37℃ x 10min |
| PR | Ventana | 1E2 | RTU | RT x 24min | BenchMark Ultra | CC1（pH8.2） x 41min | Ventana Multimer | 37℃ x 8min | DAB | 37℃ x 8min |
|  | DAKO | PgR636 | RTU | RT x 20min | DAKO Omnis | Target Retrieval Solution (pH6.1) x 70min | DAKO Envision+ Dual link system-HRP | 37℃ x 20min | DAB | 37℃ x 10min |
|  | Leica | 16 | RTU | 37℃ x 15min | Leica Bond III | ER2 x 20min | Leica Polymer | 37℃ x 8min | DAB | 37℃ x 10min |
| Ki-67 | Ventana | 30-9 | RTU | 37℃ x 16min | BenchMark Ultra | CC1（pH8.2） x 36min | Ventana Multimer | 37℃ x 8min | DAB | 37℃ x 8min |
|  | Maxim | MIB-1 | RTU | 37℃ x 20min | BenchMark Ultra | Target Retrieval Solution (pH6.1) x 36min | Ventana Multimer | 37℃ x 8min | DAB | 37℃ x 8min |
|  | Leica | K2 | RTU | 37℃ x 15min | Leica Bond III | ER2 x 10min | Leica Polymer | 37℃ x 8min | DAB | 37℃ x 8min |

RTU: Ready to use; RT: Room temperature.

**Supplementary-Fig 1** Observed agreement by different antibodies among 3 RCs and the reference value. The y-axis represents the percentage of positively stained tumour cells. RCs: Revising centres


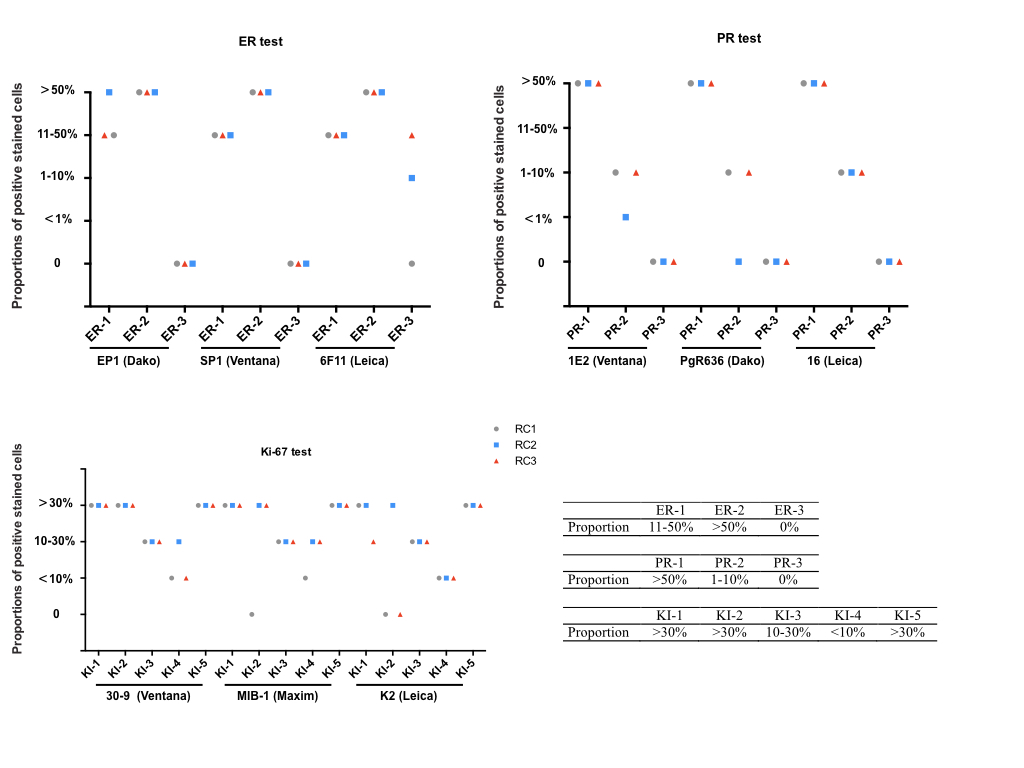

Supplement: Supplementary file 1 — Additional file 1: Table S1. Standardized IHC staining procedures of RCs. Figure S1. Observed agreement between 3 RCs and the reference value. Three RCs, the Department of Pathology, West China Hospital, Sichuan University, the Department of Pathology of Peking Union Medical College Hospital and the Shanghai Cancer Center of Fudan University, stained the slides by standardized procedures using three kinds of antibodies. As all RCs obtained the same results, the proportions of tumour nuclei positive for ER-1, ER-2 and ER-3 were 11–50%, > 50 and 0%, respectively. For the PR tests, the reference values were > 50% for PR-1, 1–10% for PR-2 and 0% for PR-3. For the Ki-67 tests, the reference values were > 30% for KI-1, KI-2 and KI-5; 10–30% for KI-3; and < 10% for KI-4. RCs: revising centres [file 12885_2019_6210_MOESM1_ESM.docx]
